# Sex-based Differences in the Association between Body Composition and Incident Fracture Risk in Koreans

**DOI:** 10.1038/s41598-017-06386-7

**Published:** 2017-07-20

**Authors:** Jung Hee Kim, A. Ram Hong, Hyung Jin Choi, Eu Jeong Ku, Nam H. Cho, Chan Soo Shin

**Affiliations:** 1 Department of Internal Medicine, Seoul National University College of Medicine, Seoul, Republic of Korea; 2 Department of Anatomy, Seoul National University College of Medicine, Seoul, Republic of Korea; 3 Department of Internal Medicine, Chungbuk National University College of Medicine, Cheongju Si, Republic of Korea; 4 Department of Preventive Medicine, Ajou University School of Medicine, Suwon, Republic of Korea; 5 Department of Molecular Medicine and Biopharmaceutical Sciences, Graduate School of Convergence Science and Technology, Seoul National University, Seoul, Republic of Korea

**Keywords:** Bone, Muscle

## Abstract

The relative contribution of lean mass and fat mass on bone health is inconclusive. We investigated the relative contributions of lean and fat masses on fragility fracture risk in Korean men and women. This was an ongoing prospective community-dwelling cohort study at Ansung beginning in 2001, which included 2,189 men and 2,625 women over 40 years old. Study subjects were classified into the following four groups according to lean mass (LM)/height^2^ and percentage fat mass (PF). Clinical fragility fracture events were assessed at baseline and biennially using self-reported questionnaires. During a median follow-up of 9.4 years, 77 (3.5%) men and 203 (7.7%) women experienced at least one incident fracture. In Cox proportional hazard models, men with low LM under normal and high PF had a 2.16 and 2.59- fold higher risk for fragility fractures than normal ones even after adjusting for covariates. However, in women, low LM or high FM was not associated with fracture risk. We demonstrated sex-based differences in the association of body composition and incident fracture risk in Koreans aged over 40 years during a 10-year follow-up duration. Maintaining muscle mass in men is vital to maintaining bone health and preventing fragility fractures in Koreans.

## Introduction

Body mass index is known to be positively associated with bone mineral density (BMD) in both sexes. Epidemiological studies have demonstrated that high BMI or body weight increases BMD and reduces fragility fractures, while low BMI or body weight is a risk factor for fragility fractures^1, 2^. However, muscle, fat, and bone each contribute to the body mass. Thus, a high BMI may be the result of a relatively higher lean mass (LM) with lower fat mass (FM) or a higher FM with lower LM.

The relative contributions of LM and FM on bone health are controversial. Some studies have proposed that LM plays a pivotal role in bone health in men and pre- and post-menopausal women^3^, while other studies have reported that fat mass is a key determinant of BMD in postmenopausal women^4, 5^. LM positively affects BMD through mechanical loading, muscle contraction, and reduced fall risk^3, 6^. FM also positively influences BMD directly by gravitational loading and the aromatization of androgens to estrogens^7^. However, the positive association of FM with BMD must be considered in the context of the close association between obesity and cardiovascular risk. Undoubtedly, low BMI is an important risk factor for osteoporosis and fragility fractures, but it is uncertain whether high BMI is always protective against osteoporosis and fragility fractures^8, 9^. A high BMI due to excess FM increases the impact force during falling and the severity of fractures. As an endocrine organ, fat produces inflammatory cytokines activating osteoclastogenesis^10^. Hence, FM may have a dual effect on bone health and fragility fractures, whereas LM seems to be beneficial for bone health.

The delineation of the roles of LM and FM is difficult due to their strong intercorrelation. Most prior studies were carried out by the linear regression analysis, inevitably allowing for the collinearity of variables. In addition, differences in age, sex, and ethnicity lead to different results. Furthermore, few studies have assessed the direct effect of body composition on fracture risk. Most studies were cross-sectional studies in a single sex, which designated BMD as the primary outcome rather than fractures.

In the present study, we aimed to elucidate the relative contributions of LM and FM on incident fracture risk by group analysis and assess the sex-based differences in the relationship between body composition and incident fracture risk in a community-dwelling prospective cohort study.

## Results

Baseline characteristics of men and women according to body composition are shown in Tables 1 and 2. Mean age was 55.5 years in men and 55.4 years in women. BMI was 23.7 ± 3.0 kg/m^2^ in men and 25.0 ± 3.4 kg/m^2^ in women. Compared with the normal group, men with low LM under normal and high PF were older and shorter. BMI and FM were lowest in men with low LM among the four groups. Men with low LM and high PF were less physically active and had lower SoS at the radius than men with normal or low LM. Of 2,425 women, 70.3% (n = 1,859) were postmenopausal. Women with low LM and high PF were older and shorter than the normal group. BMI and FM were highest in women with high PF and lowest in women with low LM. Women with low LM and high PF were less physically active. SoS at the radius was lowest in women with low LM and high PF. Subjects with high PF under normal or low LM had more chronic diseases than the normal group.

**Table 1:** Baseline characteristics of men according to body composition (n = 2,189).

	Normal LM Normal PF	Low LM Normal PF	Normal LM High PF	Low LM High PF	*P* Value	*Post hoc*
n	1051	262	778	98		
Age (years)	53.7 ± 8.5	59.6 ± 8.2	55.8 ± 8.5	61.6 ± 7.6	<0.001	a,b,c
Height (cm)	166.9 ± 5.6	163.9 ± 6.2	165.2 ± 5.7	162.4 ± 5.8	<0.001	a,b,c
Weight (kg)	64.5 ± 7.5	51.5 ± 5.3	71.8 ± 8.5	58.4 ± 6.9	<0.001	a,b,c
Weight change (kg/year)	0.02 ± 0.52	−0.05 ± 0.52	−0.17 ± 0.56	−0.14 ± 0.69	<0.001	b
BMI (kg/m^2^)	23.1 ± 2.0	19.1 ± 1.2	26.3 ± 2.2	22.1 ± 1.8	<0.001	a,b,c
Lean mass (kg)	50.3 ± 5.5	40.4 ± 3.8	50.1 ± 5.7	39.6 ± 3.8	<0.001	a,c
LM/height^2^(kg/m^2^)	18.0 ± 1.3	15.0 ± 0.8	18.3 ± 1.3	15.0 ± 1.0	<0.001	a,b,c
Fat mass (kg)	11.3 ± 2.9	8.5 ± 2.1	18.9 ± 3.7	16.0 ± 4.0	<0.001	a,b,c
Percentage fat mass (%)	17.3 ± 3.3	16.5 ± 3.2	26.2 ± 3.2	27.3 ± 4.9	<0.001	a,b,c
Physical activity (MET-h/day)	54.1 ± 21.9	56.9 ± 22.8	50.0 ± 22.6	45.5 ± 24.8	<0.001	b,c
SoS at radius (m/s)	4186 ± 146	4191 ± 156	4151 ± 169	4134 ± 150	<0.001	b,c
Regular exercise	322 (30.6)	44 (16.8)	266 (34.2)	25 (25.5)	<0.001	
History of smoking	862 (82.0)	223 (85.1)	618 (79.4)	78 (79.6)	0.184	
History of drinking	807 (76.8)	200 (76.3)	588 (75.6)	76 (77.6)	0.931	
Chronic diseases	315 (30.0)	81 (30.9)	298 (38.3)	41 (41.8)	<0.001	
Family history of fracture	28 (2.7)	0 (0.0)	13 (1.7)	2 (2.0)	0.041	
History of fracture	13 (1.2)	4 (1.5)	13 (1.7)	3 (3.1)	0.522	
Incident fracture	34 (3.2)	16 (6.1)	18 (2.3)	9 (9.2)	<0.001	

Values are means ± SD or n(%). LM, lean mass; PF, percentage body fat; BMI, body mass index; MET, metabolic equivalent; SoS, speed of sound. *Post hoc* analysis for continuous variables using the Bonferroni test (mean difference between two groups): a, normal LM/normal PF vs. low LM/normal PF; b, normal LM/normal PF vs. normal LM/high PF; c, normal LM/normal PF vs. low LM/high PF. A chi-square test was performed for categorical variables. Weight change (in kg) was calculated as the difference between weight at the last follow-up and that at the first visit divided by the follow-up years. Incident fractures were documented during the follow-up period from 2000 to 2012.

**Table 2:** Baseline characteristics of women according to body composition (n = 2,625).

	Normal LM Normal PF	Low LM Normal PF	Normal LM High PF	Low LM High PF	*P* Value	*Post hoc*
n	1238	337	933	117		
Menopause	782 (63.2)	263 (78.0)	713 (76.4)	101 (86.3)	<0.001	
Age (years)	53.6 ± 8.8	57.9 ± 9.1	56.4 ± 8.5	59.0 ± 8.4	<0.001	a,b,c
Height (cm)	153.9 ± 5.5	151.7 ± 5.8	151.9 ± 5.2	149.2 ± 5.5	<0.001	a,b,c
Weight (kg)	57.2 ± 6.3	46.9 ± 5.1	64.8 ± 7.8	52.8 ± 4.7	<0.001	a,b,c
Weigh change (kg/year)	−0.06 ± 0.48	0.01 ± 0.46	−0.25 ± 0.60	−0.19 ± 0.51	<0.001	b,c
BMI (kg/m^2^)	24.1 ± 2.1	20.3 ± 1.70	28.1 ± 2.6	23.7 ± 1.5	<0.001	a,b
Lean mass (kg)	38.7 ± 4.0	32.4 ± 3.1	38.6 ± 4.2	31.3 ± 3.1	<0.001	a,c
LM/height^2^(kg/m^2^)	16.3 ± 1.1	14.1 ± 0.6	16.7 ± 1.2	14.0 ± 0.9		a,b,c
Fat mass (kg)	16.1 ± 3.4	12.4 ± 3.1	24.0 ± 4.3	19.4 ± 3.1	<0.001	a,b,c
Percentage fat mass (%)	28.0 ± 4.1	26.1 ± 4.9	36.8 ± 3.1	36.7 ± 4.0	<0.001	a,b,c
Physical activity (MET-h/day)	49.5 ± 22.9	45.9 ± 23.1	44.9 ± 23.1	38.1 ± 23.0	<0.001	a,b,c
SoS at radius (m/s)	4198 ± 189	4167 ± 205	4161 ± 198	4139 ± 192	<0.001	a,b,c
Regular exercise	254 (20.5)	46 (13.6)	199 (21.3)	32 (27.4)	0.004	
History of smoking	56 (4.5)	26 (7.7)	54 (5.8)	4 (3.4)	0.085	
History of drinking	324 (26.2)	61 (18.1)	206 (22.1)	22 (18.8)	0.005	
Chronic diseases	422 (34.1)	79 (23.4)	444 (47.6)	51 (43.6)	<0.001	
Family history of fracture	44 (3.6)	13 (3.9)	27 (2.9)	4 (3.4)	0.795	
History of fracture	77 (6.2)	22 (6.5)	53 (5.7)	7 (6.0)	0.936	
Incident fracture	79 (6.4)	31 (9.2)	82 (8.8)	11 (9.4)	0.107	

Values are means ± SD or n(%). LM, lean mass; PF, percentage body fat; BMI, body mass index; MET, metabolic equivalent; SoS, speed of sound. *Post hoc* analysis for continuous variables using the Bonferroni test (mean difference between two groups): a, normal LM/normal PF vs. low LM/normal PF; b, normal LM/normal PF vs. normal LM/high PF; c, normal LM/normal PF vs. low LM/high PF. A chi-square test was performed for categorical variables. Weight change (in kg) was calculated as the difference between weight at the last follow-up and that at the first visit divided by the follow-up years. Incident fractures were documented during the follow-up period from 2000 to 2012.

During a follow-up duration of 9.4 years, 77 (3.5%) men and 203 (7.7%) women experienced at least one incident fracture. The numbers of fracture were 85, 66, 50, 49, and 44 at each wave. Of study subjects, 192 subjects had a previous history of fractures. We compared baseline risk factors and body composition between subjects with and without incident fragility fractures (Table 3). Postmenopausal women tended to experience more fragility fractures. Age was different between groups only in women, whereas BMI was lower in the fracture group than in the non-fracture group only in men. Women with incident fractures had lower height than those without fractures but similar weight. LM was lower in men and women with fractures than in those without fractures, but FM was significantly different only in men. Physical activity was not different between fracture and non-fracture groups. SoS at the radius was lower in women with fractures than in those without fractures. Men and women with incident fractures were likely to have experienced fragility fractures in the past.

**Table 3:** Baseline risk factors and body composition in men and women without and with incident fragility fractures.

	Men			Women		
	No fracture	Fracture	*P* value	No fracture	Fracture	*P* value
n	2,112	77		2,422	203	
Menopause				1,683 (69.5)	176 (86.7)	<0.001
Age (years)	55.5 ± 8.8	57.0 ± 8.4	0.130	55.1 ± 9.0	59.2 ± 7.5	<0.001
Height (cm)	165.7 ± 5.8	165.7 ± 5.8	0.980	152.8 ± 5.6	151.6 ± 5.3	0.003
Weight (kg)	65.4 ± 9.9	61.2 ± 8.8	<0.001	58.5 ± 8.9	57.5 ± 8.5	0.140
Weigh change (kg/year)	−0.06 ± 0.55	−0.03 ± 0.53	0.615	−0.12 ± 0.53	−0.20 ± 0.51	0.050
BMI (kg/m^2^)	23.8 ± 3.0	22.2 ± 2.8	<0.001	25.0 ± 3.4	25.0 ± 3.3	0.945
Lean mass (kg)	48.7 ± 6.5	46.1 ± 6.5	0.001	37.6 ± 4.7	36.5 ± 4.1	0.001
Fat mass (kg)	13.9 ± 5.1	12.4 ± 4.6	0.009	18.6 ± 5.6	18.8 ± 5.7	0.640
Percentage fat mass (%)	20.8 ± 5.6	19.9 ± 5.9	0. 0.165	31.2 ± 5.9	32.0 ± 6.0	0.057
Physical activity (MET-h/day)	52.6 ± 22.6	51.2 ± 23.3	0.609	46.9 ± 23.2	46.9 ± 22.9	0.994
SoS at radius (m/s)	4172 ± 157	4170 ± 153	0.928	4181 ± 196	4147 ± 194	0.018
Regular exercise	635 (30.1)	22 (28.6)	0.779	499 (20.6)	32 (15.8)	0.099
History of smoking	1716 (81.2)	65 (84.4)	0.484	128 (5.3)	12 (5.9)	0.703
History of drinking	1617 (76.6)	54 (70.1)	0.192	564 (23.3)	49 (24.1)	0.783
Chronic disease	700 (33.1)	35 (45.5)	0.025	910 (37.6)	86 (42.4)	0.177
Family history of fracture	40 (1.9)	3 (3.9)	0.214	80 (3.3)	8 (3.9)	0.628
History of fracture	29 (1.4)	4 (5.2)	0.007	130 (5.4)	29 (14.3)	<0.001

Values are means ± SD or n(%). LM, lean mass; PF, percentage body fat; BMI, body mass index; MET, metabolic equivalent; SoS, speed of sound. Weight change (in kg) was calculated as the difference between weight at the last follow-up and that at the first visit divided by the follow-up years. Incident fractures were documented during the follow-up period from 2000 to 2012.

**Figure 1:**
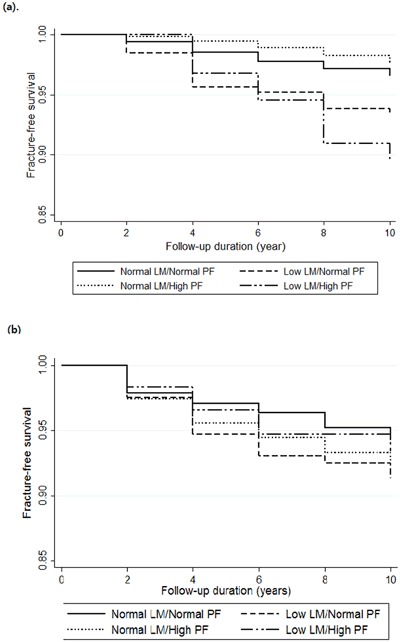
Event-free survival curve for fragility fracture according to body composition in (**a**) men and (**b**) women.

Cox proportional hazard models for fragility fractures were analyzed for the group with 1) Normal LM/Normal PF, 2) Low LM/Normal PF, 3) Normal LM/High PF, 4) Low LM/High PF (Table 4, Table 5 and Fig. 1). After 10-year follow-up, the fracture-free survival rates in men were 0.96, 0.93, 0.97, 0.88 in Normal LM/Normal PF, Low LM/Normal PF, Normal LM/High PF, and Low LM/High PF group while the fracture-free survival rates at 10-year were 0.93, 0.89, 0.90, 0.89 in women, respectively. In men, the group with low LM under normal or high PF had a 2.2 and 2.6-fold higher risk for fragility fractures than the normal group even after adjusting for age, height, weight change, physical activity, speed of sound at the radius, regular exercise, history of smoking, history of drinking, chronic disease, family history of fracture, and previous history of fracture (HR [95% CI] = 2.16 [1.13–4.16] and 2.59 [1.13–5.95], respectively). However, men with high PF did not have an increased risk for fragility fractures compared with normal subjects. Presence of chronic disease and history of fractures significantly predicted the fragility fracture risk. In sensitivity analysis including both fragility and non-fragility fractures, low LM under normal or high PF was not related with all fracture risk (Table 6). This implied that men with low LM under normal or high FM were at higher risk for only fragility fracture. On the other hand, in women, LM or FM did not affect the fragility fracture risk. Women with high PF had a higher risk for fragility fracture in unadjusted model, but after adjusting for age, the significance was lost. Age and history of fracture was the only significant risk factor for fragility fracture. We stratified the whole women group into two groups according to menopausal status (data not shown). However, there was no difference between two groups, and we showed the result altogether.

**Table 4:** Cox proportional hazard models for fragility fractures in men according to body composition.

	Unadjusted model	Model 1	Model 2
Normal LM/Normal PF	Reference	Reference	Reference
Low LM/Normal PF	2.25 (1.21–4.19)	2.12 (1.12–4.04)	2.16 (1.13–4.16)
Normal LM/High PF	0.73 (0.41–1.33)	0.74 (0.40–1.35)	0.71 (0.38–1.31)
Low LM/High PF	3.18 (1.46–6.94)	2.97 (1.31–6.73)	2.59 (1.13–5.95)
Age (per year)		1.01 (0.98–1.04)	1.01 (0.98–1.04)
Height (per cm)		1.01 (0.97–1.05)	1.00 (0.96–1.05)
Weight change (per kg/year)		1.11 (0.72–1.73)	1.23 (0.79–1.90)
Physical activity (MET-h/day)			0.99 (0.98–1.01)
SoS at radius(m/s)			1.00 (0.99–1.00)
Regular exercise			0.94 (0.55–1.60)
History of smoking			1.22 (0.63–2.35)
History of drinking			0.69 (0.41–1.16)
Chronic disease			1.74 (1.07–2.83)
Family history of fracture			2.37 (0.73–7.69)
History of fracture			3.39 (1.20–9.61)

Data are shown as HRs (95% CI). LM, lean mass; PF, percentage body fat; Model 1, adjusted for age, height and weight change; Model 2, additionally adjusted for physical activity, speed of sound at radius, regular exercise, history of smoking, history of drinking, chronic diseases, family history of fracture, and history of fracture.

**Table 5:** Cox proportional hazard models for fragility fractures in women according to body composition.

	Unadjusted model	Model 1	Model 2
Normal LM/Normal PF	Reference	Reference	Reference
Low LM/Normal PF	1.39 (0.89–2.16)	1.14 (0.73–1.78)	1.14 (0.72–1.79)
Normal LM/High PF	1.49 (1.07–2.05)	1.26 (0.90–1.75)	1.33 (0.95–1.87)
Low LM/High PF	1.27 (0.61–2.63)	0.95 (0.45–2.01)	1.03 (0.49–2.19)
Age (per year)		1.04 (1.01–1.06)	1.04 (1.01–1.07)
Height (per cm)		1.00 (0.97–1.03)	1.00 (0.97–1.03)
Weight change (per kg/year)		0.89 (0.67–1.17)	0.89 (0.67–1.18)
Menopause		1.63 (0.94–2.84)	1.68 (0.96–2.92)
Physical activity (MET-h/day)			1.00 (0.99–1.01)
SoS at radius(m/s)			1.00 (1.00–1.01)
Regular exercise			0.88 (0.59–1.30)
History of smoking			1.03 (0.56–1.87)
History of drinking			1.32 (0.93–1.86)
Chronic disease			0.94 (0.69–1.28)
Family history of fracture			1.65 (0.80–3.42)
History of fracture			1.99 (1.29–3.09)

Data are shown as HRs (95% CI). LM, lean mass; PF, percentage body fat; Model 1, adjusted for age, height, weight change and menopausal status; Model 2, additionally adjusted for physical activity, speed of sound at radius, regular exercise, history of smoking, history of drinking, chronic diseases, family history of fracture, and history of fracture.

**Table 6:** Cox proportional hazard models for all incidental fractures according to body composition.

Men	Normal LM Normal PF	Low LM Normal PF	Normal LM High PF	Low LM High PF
All fracture rate	66 (8.9)	44 (9.3)	59 (9.5)	17 (7.9)
HR (95% CI)	1	1.05 (0.72–1.54)	1.10 (0.77–1.56)	0.59 (0.52–1.52)
**Women**	**Normal LM Normal PF**	**Low LM Normal PF**	**Normal LM High PF**	**Low LM High PF**
All fracture rate	75 (7.7)	61 (10.1)	71 (8.9)	21 (8.5)
HR (95% CI)	1	1.30 (0.93–1.83)	1.12 (0.81–1.55)	1.07 (0.66–1.73)

Data are shown as HRs (95% CI).

## Discussion

We demonstrated sex-based differences in the association between body composition and incident fracture risk in Koreans aged over 40 years during a follow-up duration of 10 years. In men, low LM or high PF was the most important factor determining fragility fracture risk. On the other hand, neither low LM nor high PF was related with fragility fracture risk among women.

Only a few longitudinal studies have investigated the effect of body composition on fractures^5, 11–16^. Low LM may increase the risk of osteoporotic fractures directly or through negative effects on the BMD^14^. Muscle mass increases BMD by mechanical loading and the action of muscular strain on osteocytes^17^. Conversely, a loss of muscle mass contributes to declining muscle strength and physical performance, which leads to higher fall risk^6^. In an elderly Chinese cohort study with men aged over 65 years (n = 2,000), there was a significant association between low LM and the risk of fracture (HR = 1.87) independent of BMD and covariates. Moreover, the combination of low LM and osteoporosis further increased the risk of fractures by 3.5 times^14^. In 4,000 community-dwelling Chinese men and women aged over 65 years, low LM was associated with all fracture types (HR = 1.87) and hip fractures (HR = 2.67) in men but not in women after adjustments for age and femoral BMD^15^. Similarly, our study revealed that low LM itself increased fracture risk by 2.5-fold after adjustment for peripheral BMD (QUS at the radius) and risk factors only in men. In contrast to the present study, the above-mentioned studies defined sarcopenia using appendicular muscle mass and muscle strength instead of whole body LM^14, 15^. Whole body LM includes trunk LM in addition to appendicular. Thus, in our study, whole body LM may have a stronger loading effect on bones, which results in a high risk of fractures in men with low LM. Women with low LM in our study did not have an increased fragility fracture risk. The sex-based differences in the effect of sarcopenia on fractures have also been reported in previous studies. Taaffe *et al*. reported that LM was the dominant determinant for BMD in men but not in women^4^. A recent meta-analysis has demonstrated that the effect of LM on BMD is more robust in men than in women^3^. Men have the smaller proportion of fat mass compared with women. In terms of the mechanical loading effect of body composition, lean mass may have a bigger impact on bone mass than fat mass in men. In addition, muscle strength derived from muscle mass may affect the fracture risk in men than in women.

Previous studies have reported the relationship between obesity and fracture, but the definition of obesity was based mainly on the BMI values, which included both LM and FM. Thus, to determine the role of obesity on bone health, we needed to separate FM from BMI. Whether FM increases or decreases the fracture risk has not been determined previously. Like LM, increased FM also imposes a mechanical loading on bones, so bones may become stronger to tolerate the stress. Moreover, an aromatization of androgens to estrogens occurs in adipose tissue, which increases bone strength^7^. However, adipose tissues also secrete inflammatory cytokines such as interleukin-6 and tumor necrosis factor-alpha, which may stimulate osteoclastogenesis leading to bone resorption^10^. Independently of BMD, high FM may protect against fracture by greater soft tissue padding, whereas it may also augment the risk though higher fall risk and greater impact forces when falling^8, 9, 13, 18^. However, in women, the diverse effect of fat mass on fracture risk can be exaggerated compared with that in men. There were only a few previous reports regarding the association between FM and fracture risk. Previous studies failed to demonstrate the significant association between FM and fracture risk in men^5, 19^. In our study, men with high PF were not protected against fragility fractures. Furthermore, men with low LM and high PF showed a higher fracture risk than men with low LM and normal PF, which implied that high PF was an additional risk factor for fragility fractures in men. In a prospective cohort study, older women with higher FM had a lower risk for hip fractures, but for any or non-hip fracture risk, a nonlinear negative association was observed above 35% of PF^5^. In this regard, women in our study with PF over 33% did not show a lower risk for fragility fracture. In a large population of nearly 8,000 French women over 75 years, the hip fracture risk increased by 40% per standard deviation decline in FM^12^. This EPIDOS prospective study included high-risk women aged over 75 years during an average 2-year follow-up. The women in this previous study were much older than those in our study (over 40 years), and the follow-up duration was much shorter than ours (10 years). As predictors for fracture risk in the women in our study, age and history of fracture were the significant factors, and the age difference may have led to the different results.

The discrepancy between our study and previous studies may be derived from the analytic methods. The previous studies assessed the effect of LM and FM individually and then further adjusted for height or weight. However, LM and FM are highly correlated, and different body compositions may affect fracture risks differently even with the same bodyweight. In light of this, out study is noteworthy in that we analyzed LM and FM simultaneously using a group analysis. Due to different body size, we used total LM adjusted by height squared and PF. There were also several reports that after adjusting for height and weight, FM was negatively correlated with BMD^18, 20, 21^. Those studies implied that if FM was not compensated by the mechanical loading effect, it could be harmful to bone health.

In the present study, we determined that sex was another important factor in determining the connection between body composition and incident fracture risk. Ho-Pham *et al*. demonstrated that the effect of LM on BMD is stronger in men than in women in a meta-analysis^3^. These results were in agreement with our results. However, FM appears to be positively associated with BMD, but after adjustment for total body size, it is negatively associated. Our findings supported this opinion by group analysis according to body composition.

Our study has several strengths. The major strength was a 10-year prospective cohort study in a relatively large sample size in middle-aged to older Korean men and women. We set the primary outcome variable to be fragility fractures, which is the most solid outcome variable to reflect bone health. Previous studies investigated the relationship between body composition and BMD rather than fractures^3, 20^. Low BMD is an important predictor for fracture risk, so the association with BMD is an important clue to assess the relationship between body composition and fracture. However, fracture risk also includes non-BMD factors such bone strength and falling risk. In addition, we considered LM and FM simultaneously, which occurs in real practice. Moreover, we discriminated FM from BMI as a definition of obesity, which allowed for assessment of FM in predicting fracture risk.

There are inevitable limitations in the present study. Compared with DXA or CT, the reliability of BIA is controversial. The accuracy of BIA is dependent on the environmental conditions during measurement such as temperature or humidity, and the individual’s hydration status^22^. However, since BIA is a simple, fast, noninvasive, and radiation-free tool, it was recommended for the diagnosis of community sarcopenia and validated in Asians^23, 24^. We measured total LM instead of appendicular LM, which is different from current sarcopenic indices, and our data cannot be directly compared with data from other studies^25, 26^. Moreover, the cut-off value of sarcopenia and obesity were defined within our population, thus external validation was necessary. The fat distribution was not measured, so the differences in the effects of subcutaneous and visceral fat on BMD were not assessed^18, 21^. The SoS at the radius using QUS was not as accurate as that obtained by using central DXA, although previous studies have used QUS instead of DXA^27, 28^. Thus, there was no difference noted in the SoS between men with and without fractures. For 10 years, time-dependent covariates such as menopause should be considered. Fragility fractures were not adjudicated by medical records but self-reported. Fall risk assessment was not included in our cohort study. In addition, serum vitamin D levels were not measured in the current study, and the proportion of patients with vitamin D supplementation may be underestimated due to the use of self-reported data.

We demonstrated that low LM alone or combined with high PF was a risk factor for fragility fractures in Korean men. In women, we failed to draw conclusion regarding the body composition and fracture risk. Increasing muscle mass in men is vital to maintaining bone health and preventing fragility fractures in Koreans.

## Methods

### Ethical statement

Study procedures were performed according to institutional guidelines and approved by the ethics committee of the Korean Center for Disease Control and the institutional review board of Ajou University School of Medicine (IRB No AJIRB-CRO-07-012). Informed consent was obtained from the study subjects. The study was conducted according to the Declaration of Helsinki.

### Study subjects

**Figure 2:**
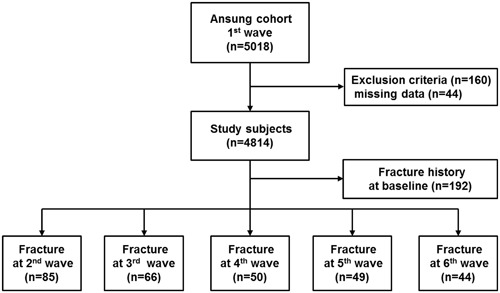
Flow diagram of study subjects.

We analyzed data from the Ansung cohort study, a part of the Korean Health and Genome Study. The Ansung cohort study is an ongoing prospective community-dwelling cohort study which was designed to investigate the epidemiology of chronic diseases in Korea. Begun in 2001, a total of 2,239 men and 2,779 women aged 40–69 years at baseline were included and examined biennially. Of these, subjects were excluded from this analysis (n = 204) if they had a history of malignancy or had received any drug that might affect bone metabolism, such as vitamin D, hormones, and medications for osteoporosis, for more than a 6-month period or within the previous 12 months. In the final analysis, 2,189 men and 2,625 women were enrolled (Fig. 2).

### Anthropometric and body composition measurement

Height and body weight were measured in light clothing at baseline. BMI was calculated as weight divided by height squared (kg/m^2^). Weight change (in kg) was calculated as the difference between weight at the last follow-up and that at the first visit. We measured body composition by using bioelectrical impedance analysis (BIA, Inbody 3.0, Biospace Co., Korea). BIA measures human body composition through tissue conductivity. Skeletal muscle is the largest component of the human body; it is an electrolyte-rich tissue with low resistance^29^. The coefficients of variation ranged from 1.8% to 2.9%. Study subjects were examined as follows: 1) standing with their arms slightly separated from the trunk and legs slightly straddled for 5–10 min and 2) grasping the handles of the machine and contacting the eight electrodes (two for each foot and each hand). When the measurements stabilized, the analyzer displayed resistance directly and immediately.

Low LM was defined as under the 20^th^ percentile of sex-specific total LM divided by height squared, which was 16.0 kg/m^2^ for men and 14.8 kg/m^2^ for women^30^. High percentage fat mass (PF) was defined as the higher sex-specific two quintiles of total PF, which was 22% for men and 33% for women^31^. According to body composition, we categorized study subjects into the following four groups: 1) normal LM and PF, 2) low LM, 3) high PF, and 4) low LM and high PF.

### Fracture events assessment

Clinical fracture events were assessed at baseline and biennially using self-reported questionnaires. Situations surrounding the fractures were also reported in the questionnaires; we excluded high-trauma fractures such as those from a car accident, fall from a height more than standing height, and severe traumas. Low-trauma fractures were defined as a fracture from a fall from standing height or less. Fragility fractures were defined as low-trauma fractures occurring at the hip, vertebrae, proximal humerus, and radius. Previous history of fractures included fragility fractures occurring before baseline examination. Incident fractures were documented during the follow-up period from 2000 to 2012.

### Quantitative Ultrasonography (QUS) Measurements

QUS measurements were made at the distal third of the radius using the Omnisense 7000 device (Sunlight Medical, Ltd., Rehovot, Israel) through a handheld probe designed for measurements of the axial speed of sound (SoS, m/s) along the surface of bone. All subjects were examined three times on their non-dominant sides and repositioned between measurements. The mean value of the three measures was defined as the final SoS value at the radius. Quality controls of the Omnisense device were performed daily using a manufacturer-provided SoS verification phantom. The mean coefficients of variation (CVs) for measurement of the radius were 0.22% for interobserver validity and 0.24% for intraobserver validity^28^. The SoS at the distal radius and total hip bone mineral density measured by dual X-ray energy absorptiometry was positively correlated (*r* = 0.246, *p* < 0.001), which was analyzed from the data from 4^th^ wave.

### Covariates

Women were classified as postmenopausal if they had experienced no menstrual bleeding in the last 12 months. Information regarding regular exercise, history of smoking, history of drinking, chronic diseases, family history of fracture, and previous history of fracture were collected at baseline by a standardized questionnaire. Regular exercise was defined as doing any type of exercise more than once per week. History of smoking and drinking included all former and current users. Chronic diseases consisted of cancer, myocardial infarction, congestive heart failure, diabetes, hypertension, osteoarthritis, and pulmonary diseases and were based on self-reported questionnaires^32^. Previous history of fractures was defined as fragility fractures occurring before the baseline examination. The Korean version of the International Physical Activity Questionnaire (IPAQ) was used to assess physical activity (PA). An average metabolic equivalent (MET) score was calculated based on Ainsworth *et al*.’s suggestions^33^: <3.0 METs for light PA, 3.0–6.0 METs for moderate PA, >6.0 METs for vigorous PA. Total PA (MET-hours/day) was defined as the sum of the daily METs of light, moderate, and vigorous PA.

### Statistical Analysis

Data were presented as mean ± standard deviation or n (%). We classified study subjects into four groups in each sex according to body composition: 1) normal LM/normal PF, 2) low LM/normal PF, 3) normal LM/high PF, and 4) low LM/high PF. An analysis of variance (ANOVA) for continuous variables and a chi-square test for categorical variables were applied to compare variables among four groups. The *Post hoc* analysis for continuous variables between two groups was performed using the Bonferroni test. In comparing baseline risk factors and body composition between subjects with and without incident fragility fractures, we used Student’s *t*-test. The survival analysis for assessing fragility fracture risk according to body composition was performed by cox proportional hazard models using covariates as age, height, weight change, physical activity, speed of sound at the radius, regular exercise, history of smoking, history of drinking, chronic disease, family history of fracture, and previous history of fracture. A *p* Value of <0.05 was considered to be significant. Statistical analyses were carried out using the PASW SPSS for Windows (version 21, SPSS Inc., Chicago, Illinois, USA) and STATA version 14.0.
