# Author Correction: Sex-based Differences in the Association between Body Composition and Incident Fracture Risk in Koreans

**DOI:** 10.1038/s41598-019-54420-7

**Published:** 2019-11-28

**Authors:** Jung Hee Kim, A. Ram Hong, Hyung Jin Choi, Eu Jeong Ku, Nam H. Cho, Chan Soo Shin

**Affiliations:** 1 Department of Internal Medicine, Seoul National University College of Medicine, Seoul, Republic of Korea; 2 Department of Anatomy, Seoul National University College of Medicine, Seoul, Republic of Korea; 3 Department of Internal Medicine, Chungbuk National University College of Medicine, Cheongju Si, Republic of Korea; 4 Department of Preventive Medicine, Ajou University School of Medicine, Suwon, Republic of Korea; 5 Department of Molecular Medicine and Biopharmaceutical Sciences, Graduate School of Convergence Science and Technology, Seoul National University, Seoul, Republic of Korea

Correction to: *Scientific Reports* 10.1038/s41598-017-06386-7, published online 20 July 2017

The original version of this Article omitted an affiliation for Jung Hee Kim. The correct affiliations for Jung Hee Kim are listed below:

Department of Internal Medicine, Seoul National University College of Medicine, Seoul, Republic of Korea

Department of Molecular Medicine and Biopharmaceutical Sciences, Graduate School of Convergence Science and Technology, Seoul National University, Seoul, Republic of Korea.

This has now been corrected in the HTML and PDF versions of this Article.

